# Controlling the three dimensional propagation of spin waves in continuous ferromagnetic films with an increasing out of plane undulation

**DOI:** 10.1038/s41598-021-00479-0

**Published:** 2021-11-01

**Authors:** Federico Montoncello, Gianluca Gubbiotti

**Affiliations:** 1 Dipartimento di Fisica e Scienze della Terra, Università degli Studi di Ferrara, 44122 Ferrara, Italy; 2 Istituto Officina dei Materiali del CNR (CNR-IOM), c/o Dipartimento di Fisica e Geologia, Università degli Studi di Perugia, 06123 Perugia, Italy

**Keywords:** Ferromagnetism, Magnetic properties and materials, Magnetic properties and materials

## Abstract

The role of three-dimensionality in a ferromagnetic medium in ruling the propagation properties of spin-waves (SW) has been one of the main focuses of the research activity in recent years. In this context, we investigate the evolution of the SW dispersion (frequency vs wave vector) induced by a progressive vertical undulation of a ferromagnetic film. The geometric undulation is taken along a single direction and is periodic with constant period, while the amplitude (differential maximum height with respect to the film thickness) is gradually increased from 0 to 60 nm. We study the characteristic modification of the internal effective field and link it to the resulting SW dispersions and spatial profile. These systems display at once features both of a planar film and a discretized medium, and the dispersion curves change not only when SWs propagate along the undulation direction, but also perpendicular to it. We discuss the geometric and magnetic conditions for having either the invariance of the SW group velocity with respect to even major changes in the undulation, or a large group velocity for some edge modes. We address a potential dual-band activity, namely the simultaneous propagation of two independent SW-signals, with separated frequency bands and disjoint oscillation regions.

## Introduction

The propagation of waves in periodically structured materials is of fundamental interest in modern physics and technology, and when spin waves (SWs) in ferromagnetic media are concerned, the corresponding research field is Magnonics^1–3^. In Magnonics, SWs play the role of information carriers, which are exploited to process and store information and operate in ultra-low-power conditions. When propagating along waveguides consisting of artificial magnetic materials with properties periodically varied in space (magnonic crystals), SWs undergo Bragg diffraction and acquire new properties, absent in bulk materials, due to the formation of frequency bands and band gaps. Since these properties depend on geometry and applied magnetic field, they can be easily designed and reconfigured. For these reasons, Magnonics is considered particularly promising for a possible beyond-CMOS technology^4^. In the last decades, the conceptual problem was just studying SW propagation, which is inherently a two-dimensional problem, since a signal travelling along a waveguide can be embedded in one- (1D) or two-dimensional (2D) space, depending on the curvature^5–9^. Hence, the research exploration has mainly involved structures displaying 1D/2D magnetization textures, either periodic^10–13^ or aperiodic^14–17^, while structures with in-plane periodicity, but inhomogeneous along the perpendicular direction, have been poorly explored so far. However, exploring the third dimension is crucial indeed, because a 3D distribution of the magnetization offers a new degree of freedom, which in general allows, from one side, to fit more functionality into a smaller space, and hence to considerably increase the density of elements in magnonic devices^18,19^, and from the other, further possibilities for the SW dynamics and transport (e.g., vertical magnon transport, nonreciprocal coupling^20–22^). More recently, the opportunity of designing 3D ferromagnetic systems where ferromagnetic layers are linked by a large number of vertical connections has been proposed in the perspective of the so-called neuromorphic networks^23–25^.

In recent times, thanks to the advances in the vertical resolution of e-beam lithography, and also in the self-assembly synthesis techniques^26^, the study of three-dimensional arrangements of nanomagnets has become more and more concrete even from the experimental point of view^18,27,28^. The third dimension has been gradually introduced in waveguides and planar nanomagnet distributions though the vertical stacking of layers with designed profile acting as a switch to allow specific, non-uniform, magnetization distributions in the plane, thanks to the coupling magnetic fields both of dipolar and exchange nature^29–31^.

In this paper, we study how spin waves behave when a planar ferromagnetic film is progressively distorted into a periodically undulated one, involving the third dimension (out-of-plane). Pilot studies have been published until now, covering either the static^32^ or dynamic^33–37^ issues, which we extend and generalize.

Our results show how the geometric out-of-plane undulation of a continuous film impacts the SW propagation, altering the phase profile and frequency/wavevector dispersion relation for the SW not only propagating along the undulation direction, but also perpendicular to it. We characterize the spin dynamics either when the film is magnetized parallel or perpendicular to the undulation direction, namely in the conditions where magnetization is uniform or non-uniform, and provide rules for the occurrence of a forbidden gap at zone boundary, and in particular showing its absence in all the cases where the magnetization is continuous across the primitive cells. In general, the SWs propagating through an undulated medium along any direction have a mixed character, since they experience periodicity only along a specific coordinate and wavevector component. To this purpose, we discuss the coexistence, in the same system, of periodic waves together with non-periodic waves, and, on the other side, of bulk waves together with edge waves when the magnetization is not uniform.

Moreover, we delineate the geometric and propagation conditions for the SW group velocity to be topologically invariant, so to be possibly used as a reference value protected against undulation alterations. On the other side, we find out how special edge modes, forming in a specific 3D structure, show a frequency bandwidth comparable to or even larger than the bulk wave ones, hence allowing an additional channel for simultaneous information delivery (dual-band action). Our findings are worth to be taken into consideration at the stage of designing the magnonic structure to tailor SW properties on demand.

## Results

### System modeling

**Figure 1:**
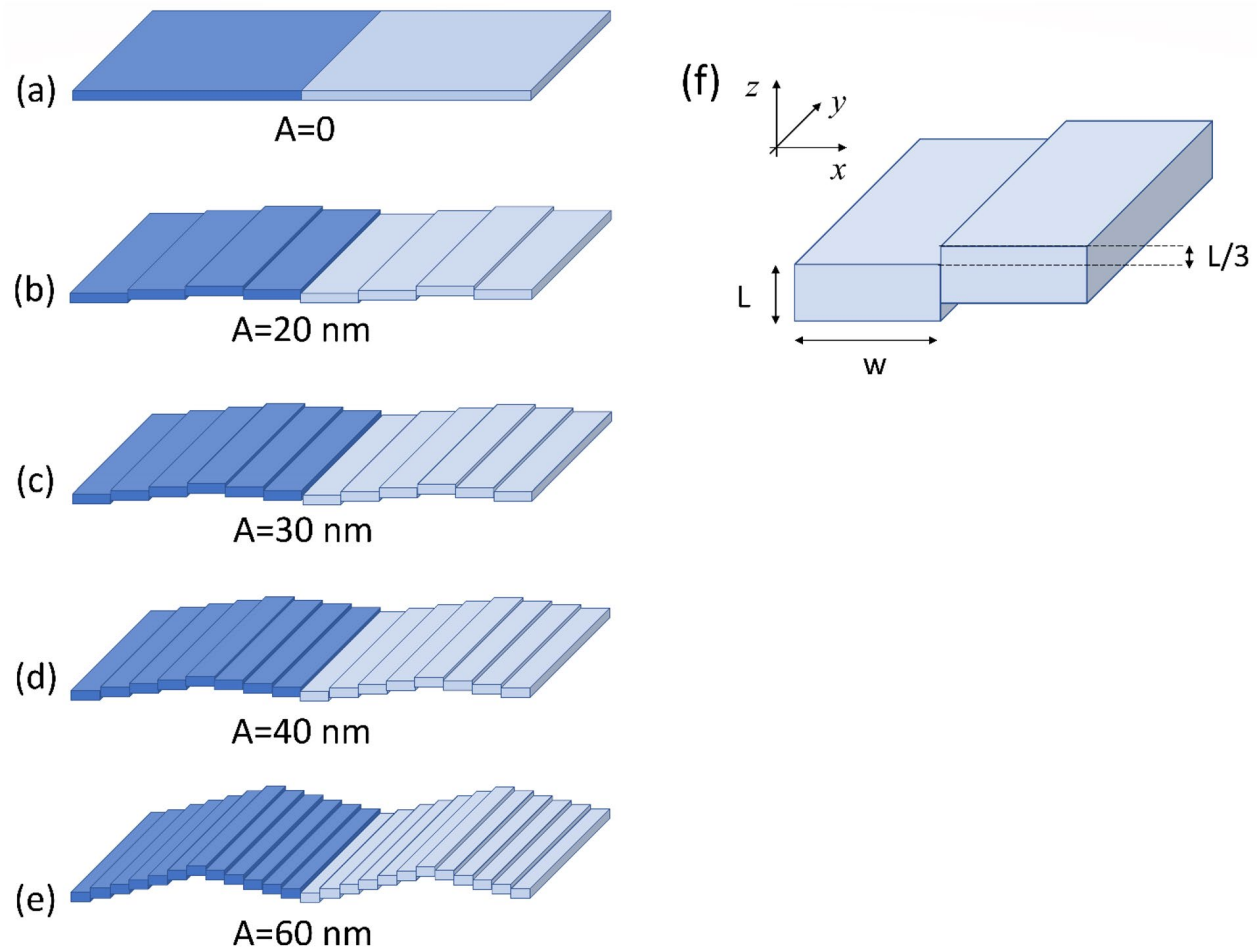
Geometric structures used in the study: **(a–e)**, in perspective, the six primitive periodic cells (darker blue) and a repetition of each (lighter blue), as an illustration of the infinite repetitions along both in-plane coordinates; the periodic cell is 720 nm wide, and the geometric undulation is attained through a number of elemental stripes of width w. **(a)** is the planar film, with w = 720 nm equal to the (virtual) periodic cell width; **(b)** 4-stripe system, with w = 180 nm; **(c)** 6-stripe system, with w = 120 nm; **(d)** 8-stripe system, with w = 90 nm; **(e)** 12-stripe system, with w = 60 nm. **(f)** The reference system and the geometric displacement h = L/3 of adjacent stripes, with constant thickness L = 30 nm and varying width w. The undulation amplitude A is specified below each panel.

As discussed in section “Methods”, we use a micromagnetic simulator to compute the equilibrium magnetic configuration, and the dynamical matrix method (DMM) to get the SW frequency and profile at any given wavevector value and direction: both calculation tools are based on the discretization of the sample into a tridimensional mesh of prisms. Given such a framework, we conceived the undulation as a sequence of infinitely long elemental stripes with thickness L = 30 nm but varying width *w* and displacement *d* along the z coordinate, depending on the geometric, out-of-plane, undulation amplitude (Fig. 1). Each stripe is made of three 10-nm thick layers, and the displacement along z between adjacent stripes is *h* = 10 nm, so that L = *3 h* and the generic displacement of any stripe, with respect to the ground (*z* = 0), is *d* = *Mh*, with integer *M*. We choose to fix the undulation period to *a* = 720 nm. Hence, if *N* is the number of stripes forming the primitive cell, the overall extent of the system along the z direction is ${d}_{N}^{MAX}=\frac{N}{2}h+L$, while the undulation amplitude is defined as $A={d}_{N}^{MAX}-L$ as shown in Table 1. In practice, the planar film has zero undulation amplitude, while the 12-stripe system attains the largest amplitude, i.e., $A=$ 60 nm.

**Table 1:** The 5 systems under investigation and the corresponding overall extent along the z direction, undulation amplitude, and width of the elemental stripe forming the primitive cell.

	*N*	Undulation amplitude $A$ (nm)	Overall extent along z ${d}^{MAX}$ (nm)	Elemental stripe width $w$ (nm)
Planar film	1	0	30	720
4-stripe system	4	20	50	180
6-stripe system	6	30	60	120
8-stripe system	8	40	70	90
12-stripe system	12	60	90	60

The magnetization distribution has peculiar features, depending on the direction of the applied field (with constant magnitude ${B}_{0}\equiv {\mu }_{0}{H}_{0}=0.1 T$) with respect to the undulation direction, which is kept along x-axis. This is because the geometric arrangement acts as a constraint for the magnetic moments, and determines a specific internal effective field ${{\mu }_{0}\mathbf{H}}_{\mathrm{eff}}$, which is the net sum of the external field (in our case, 0.1 T), and the internal dipolar and exchange fields. ${\mu }_{0}{\mathbf{H}}_{\mathrm{eff}}$ is a reference quantity, because it rules both the static distribution of the magnetization and its dynamics.

**Figure 2:**
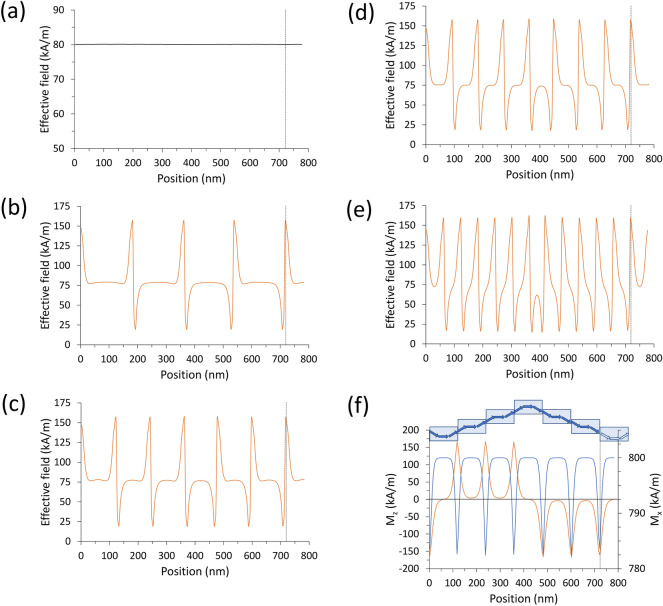
**(a–e)** Space profiles along the undulation direction of the internal effective field component parallel to the average magnetization direction: in (a), when ${\varvec{M}}\parallel {\varvec{y}}$, the profile ${H}_{eff}^{y}\left(x\right)$ is uniform independently of the undulation amplitude (from the planar film to the 12-stripe system); despite the many geometric discontinuities, due to the different heights of the adjacent stripes, ${H}_{eff}^{y}\left(x\right)$ is uniform (computational confidence within 0.05 kA/m) independently of the applied field magnitude. In **(b–e)**, when ${\varvec{M}}\parallel {\varvec{x}}$, instead, ${H}_{eff}^{x}\left(x\right)$ is highly non-uniform: **(b)** 4-stripe system; **(c)** 6-stripe system; **(d)** 8-stripe system; **(e)** 12-stripe system. In **(f)** we plot both magnetization components ${M}_{z}(x)$(orange curve, left axis) and ${M}_{x}(x)$(blue curve, right axis) of the top layer in the (representative) 6-stripe system, when ${\varvec{M}}\parallel {\varvec{x}}$; a sketch of the magnetization along the primitive cell is juxtaposed, illustrating the behavior of its out-of-plane component across the vertical sections of the 6 stripes.

If the magnetization $\mathbf{M}$ is along ***y***, i.e., perpendicular to the undulation direction, then no geometric disturbance of the magnetic moment alignment occurs, and hence the distribution is uniform ($\nabla \cdot \mathbf{M}=0$, i.e., the magnetization flux lines are parallel) and so is the internal effective field ($\nabla \cdot {\mathbf{H}}_{\mathrm{eff}}=0$, see Fig. 2a). The only change from the planar arrangement Fig. 1a to the undulated ones (b-e) is the height of the adjacent stripes along x-direction, which implies a small increase of the exchange field due to the lack of any adjacent moments at the edges of each stripe, but a considerable decrease of the total dipolar field since, on the average, the adjacent stripes (and the magnetic moments inside) are farther when misaligned than when aligned to form the planar film. Altogether, this implies a progressive increase of  ${{\mu }_{0}\mathbf{H}}_{\mathrm{eff}}$ with respect to the undulation amplitude, resulting in an overall increase of the SW frequency.

Conversely, if the magnetization $\mathbf{M}$ is along ***x***, i.e., parallel to the undulation direction, then the distribution is no more uniform, and magnetic charges form, with density ${\rho }_{M}=-\nabla \cdot {\mu }_{0}\mathbf{M}$; in turn, also the internal effective field varies along $x$: in Fig. 2b–e, in each primitive cell ${\mathbf{H}}_{\mathrm{eff}}\left(x\right)$ displays plateaux in the stripe bulk and peaks at the stripe edges, repeated for the number of stripes forming the primitive cell. In Fig. 2f we illustrate the behavior of the magnetization inside each stripe, and plot the magnetization components ${\mathrm{M}}_{\mathrm{z}}$ and ${\mathrm{M}}_{\mathrm{x}}$ along the *x* direction. Both plots exhibit peaks at the same heights, showing unambiguously that the actual lattice constant, for a unitary primitive cell, is different from the geometric lattice constant, and it corresponds to the width of the single stripe *w* This observation supports the finding, discussed in the following, that a gap opening (mode repulsion) would be possible only if the effective primitive cell was *w*, and not *a*, and hence zone boundary was $\frac{\pi }{w}$ and not $\frac{\pi }{a}$. In an ideally continuous film $w\to 0$, and no gap is ever possible, despite the nonuniformity.

### Dispersion relations and mode profiles

Through the DMM, we calculated the dispersion curves, in the five different systems (Fig. 1a–e), and the different magnetization configurations (i.e., ${\varvec{M}}\parallel {\varvec{y}}$ and ${\varvec{M}}\parallel \mathbf{x}$), for SWs propagating either perpendicular or parallel to the magnetization direction, (i.e., $\mathbf{k}\perp \mathbf{M}$ or $\mathbf{k}\parallel \mathbf{M}$). We recall that the output of DMM (i.e., the dynamic magnetization) can be interpreted in terms of Bloch waves, which, at a given time, are written as:

1 $$\delta {m}_{\mathbf{k}}\left(\mathbf{r}\right)=\delta {\tilde{m }}_{\mathbf{k}}\left(\mathbf{r}\right){e}^{i\mathbf{k}\cdot \mathbf{r}}$$

where $\delta {\tilde{m }}_{\mathbf{k}}\left(\mathbf{r}\right)$ is the cell function, of which we’ll be showing the real z-component profiles in the following, $\mathbf{r}=\left(x,y\right)$ is the in-plane position vector and $\mathbf{k}=\left({k}_{x},{k}_{y}\right)$ is the wavevector. In our calculations, we investigated only on the two main limit directions, i.e., $\mathbf{k}=\left(0,{k}_{y}\right)$ and $\mathbf{k}=\left({k}_{x},0\right)$.

**Figure 3:**
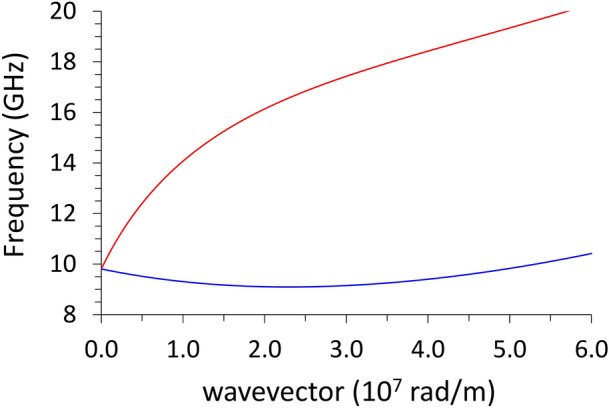
Dispersion relation for a 30-nm thick infinite film: upper curve (red) refers to the surface Damon-Eshbach mode; the lower curve (blue) refers to the volume backward mode. The magnetic parameters are the same used in the simulations.

As a first point, intending to provide a reference, we plot in Fig. 3 the analytical dispersion relation of the surface Damon-Eshbach (DE) mode $\left(\mathbf{k}\perp \mathbf{M}\right)$ and the volume backward (BA) mode $\left(\mathbf{k}\parallel \mathbf{M}\right)$ in a wide range of wavevector values. For the latter (BA), the range is enough to display both the negative slope at low wavevector (due to dipolar effects) and the parabolic rise above 2.5 × 10^7^ rad/m (due to exchange effects). The equations for the dipolar-exchange SWs are^38,39^:

2 $${\omega }_{DE}=\sqrt{\left({\omega }_{0}+{\lambda }_{ex}^{2}{\omega }_{M}{k}^{2}\right)\left[\left({\omega }_{0}+{\lambda }_{ex}^{2}{\omega }_{M}{k}^{2}\right)+{\omega }_{M}\right]+\frac{{\omega }_{M}^{2}}{4}\left(1-{e}^{-2kL}\right)}$$

and

3 $${\omega }_{BA}=\sqrt{\left({\omega }_{0}+{\lambda }_{ex}^{2}{\omega }_{M}{k}^{2}\right)\left[\left({\omega }_{0}+{\lambda }_{ex}^{2}{\omega }_{M}{k}^{2}\right)+{\omega }_{M}\frac{1-{e}^{-kL}}{kL}\right]}$$

where ${\lambda }_{ex}$ is the exchange length, which defines the range below which is not likely to have a nonuniform spin distribution, namely:

$${\lambda }_{ex}= \sqrt{\frac{2A}{{\mu }_{0}{M}_{S}^{2}}}$$

$A$ is the exchange stiffness constant, ${M}_{S}$ is the saturation magnetization and ${\omega }_{0}=\gamma {\mu }_{0}{H}_{0}$ and ${\omega }_{M}=\gamma {\mu }_{0}{M}_{S}$. In our case, ${\mu }_{0}{H}_{0}=0.1 \mathrm{T}$, $A=10 \mathrm{pJ}/\mathrm{m}$, ${M}_{S}=800 \mathrm{kA}/\mathrm{m}$, and the gyromagnetic ratio $\gamma =185 \mathrm{rad GHz}/\mathrm{T}$. With these parameters we get ${\lambda }_{ex}=5.0 \mathrm{nm}$.

**Figure 4:**
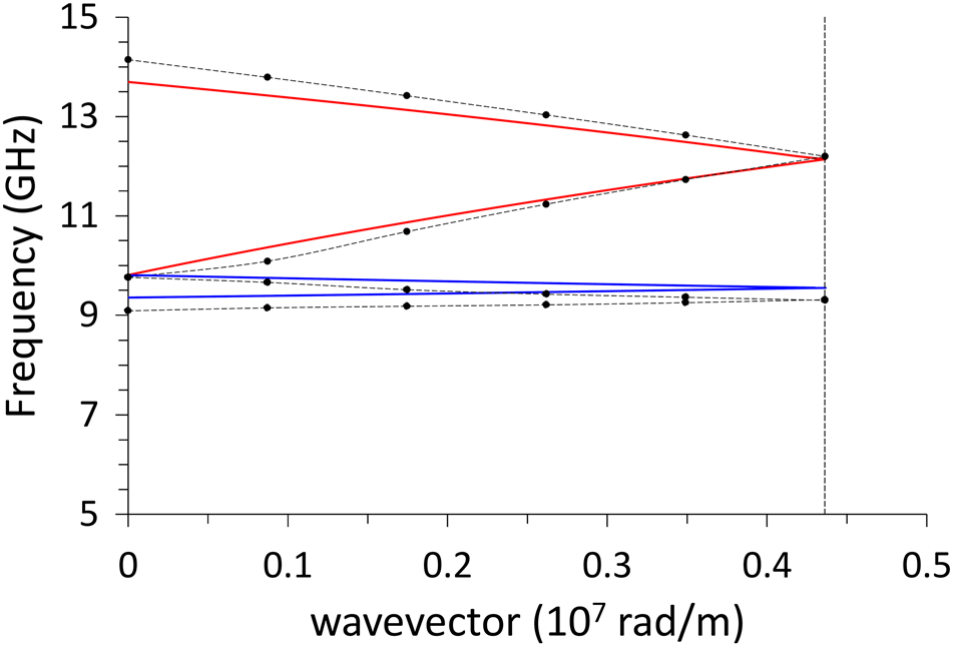
Dispersion relation (frequency vs wave vector) for the planar film, mirrored within the reduced Brillouin zone: analytical solutions (solid lines) and DMM results (symbols, dashed lines are guides for the eyes). The red curve refers to the surface Damon-Eshbach mode; the blue curve refers to the volume backward mode. The vertical dashed line, at k = 4.36 × 10^7^ rad/m is the virtual zone boundary if the primitive cell lenth was the one in Fig. 1.

Since our purpose is to compare the dispersions to the cases with periodic undulations, it is useful to fold the dispersions to the reduced Brillouin zone [0, $\frac{\pi }{a}$] (in our case a = 720 nm). Besides, in calculations, we do use periodic boundary conditions even when simulating the flat, infinite film, so we can plot in Fig. 4 both the analytical curves and the results of the calculations with the DMM: the agreement is very good, despite the completely different approaches, and the unavoidable computational errors.

As a general comment about Fig. 3, with a particular emphasis on the BA curve, we notice that the exchange contribution becomes appreciable above 2.5 × 10^7^ rad/m, where the curve slope apparently changes sign. Such a wavevector value corresponds to a wavelength of 250 nm, hence we cannot expect exchange effects for larger wavelengths.

Now we comment the results of the DMM applied to the systems of Fig. 1, concerning the effects of the undulation on the dispersions, when the magnetization is either perpendicular or parallel to the undulation direction.

### Configuration $M\parallel \mathrm{y}$

**Figure 5:**
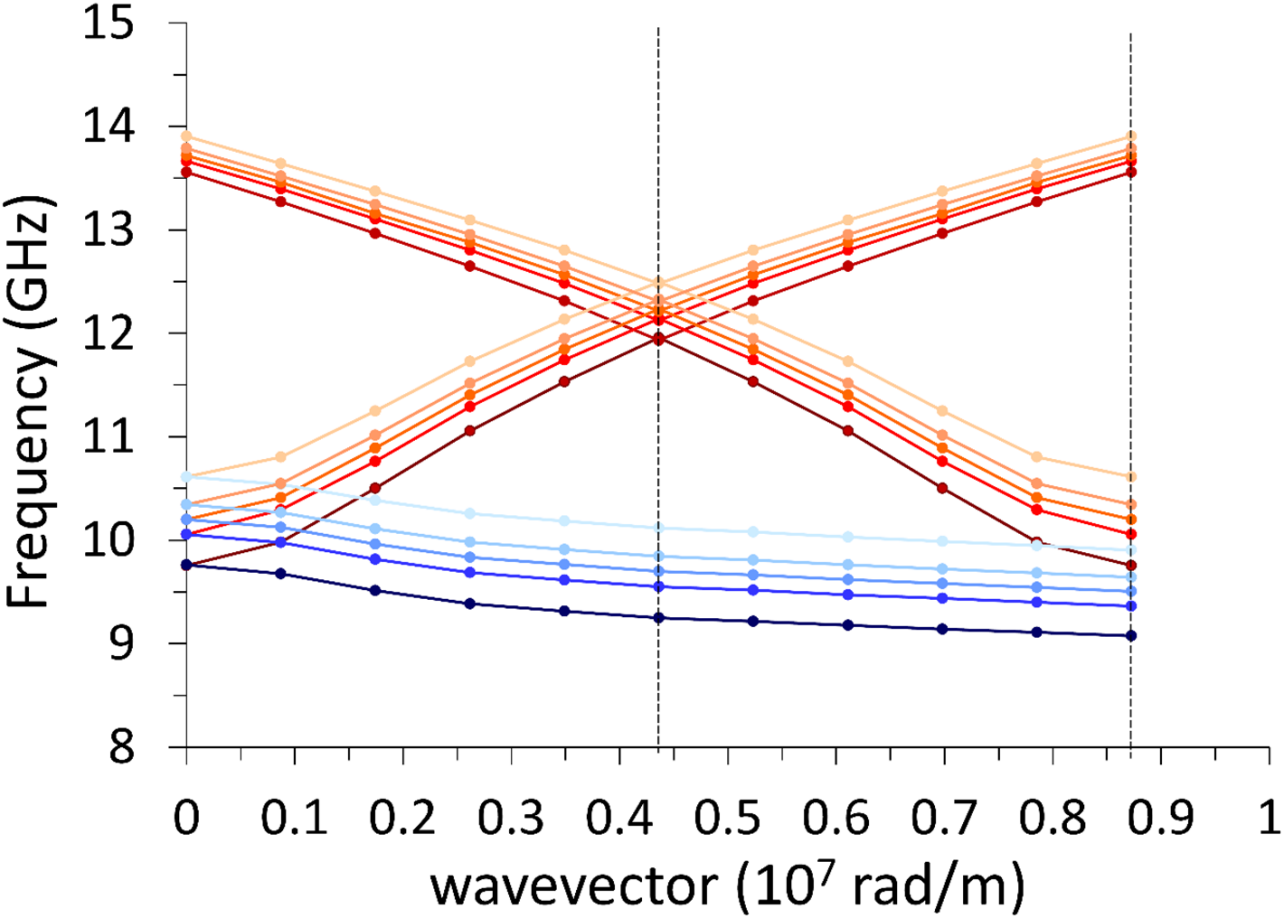
Dispersion curves in the 1st and mirrored 2nd Brillouin zone of the DE mode (red shades) and of the BA mode (blue shades), when ${\varvec{M}}\parallel {\varvec{y}}$. Lines are guides for the eyes. The vertical dashes mark the first and second zone boundaries (relevant to DE mode only). The variation of the undulation amplitude $A$ is represented by the variation of shade: the darkest is the planar film ($A=0$), the lighter is the 12-stripe system ($A=$ 60 nm). Frequency increases about 0.14 GHz every $\Delta A=$ 10 nm. The curves corresponding to the 2nd zone are less accurate, because of the finite discretization of the sample. Despite propagating along the direction where magnetization is uniform (y), the BA mode is nonetheless sensitive to the undulation amplitude changes.

**Figure 6:**
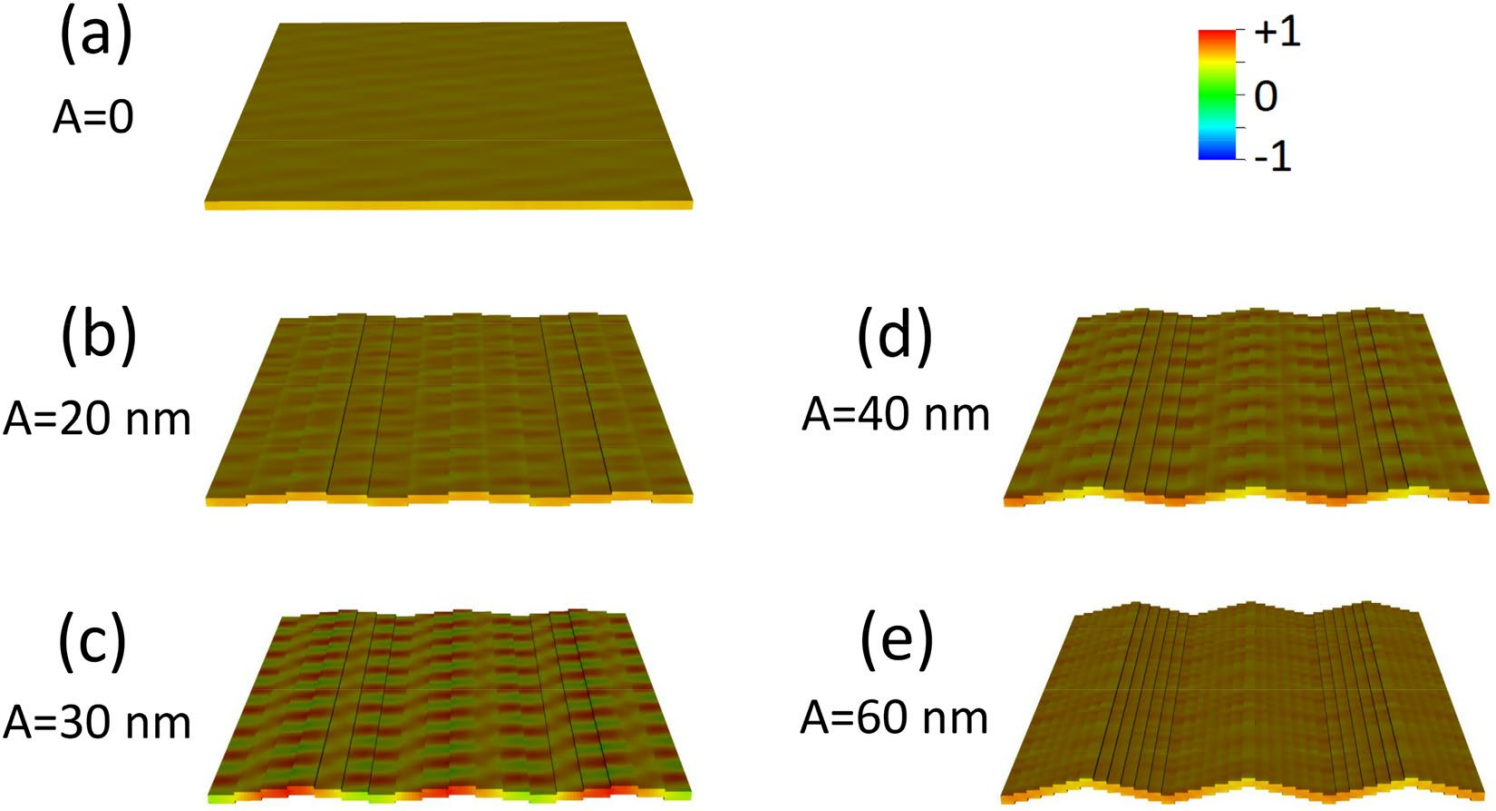
Real part of the out-of-plane (z) dynamic magnetization phase profile (colormap in arbitrary units) for k = 0, in configuration ${\varvec{M}}\parallel {\varvec{y}}$, for increasing undulation amplitude $A$, shown in perspective to highlight the involvement of the third dimension: **(a)** planar film with 30 nm thickness, i.e., . $A=$ 0; **(b)** 4-layer system, i.e. $A=$ 20 nm; **(c)** 6-layer system, i.e., $A=$ 30 nm; **(d)** 8-layer system, i.e., $A=$ 40 nm; **(e)** 12-layer system, i.e., $A=$ 60 nm. The apparent non-uniform phase profile of the cell function is an unavoidable computational effect, though the ideal result should be a uniform profile. Note that each map is a 3 × 3 repetition of the primitive cell, as an indicative extract of the ideally infinite system.

With reference to the first Brillouin zone, we observe that the increasing undulation amplitude produces an almost linear rigid shift in frequency of the dispersions, upward for DE mode, downward for the BA mode, with almost no alteration of the curvature, particularly for the BA mode (Fig. 5). Both modes have an ideal uniform cell function (Eq. 1), even though DMM output shows an artefact undulation, characteristic of the numerical calculation (Fig. 6), which is not a real effect. For either modes, at *k* = 0 the average incremental frequency shift, on increasing undulation amplitude $A$, is almost constant and equal to about $\Delta \nu /A=0.014 GHz/nm$ (which means $0.14 \mathrm{GHz}$ for every $10$-nm-increase, following Table 1). This evidence implies that the group velocity ${v}_{g}$ is almost not sensitive (i.e., is “robust”)^40^ to the exact amplitude of the undulation transverse to the propagation direction, and might be considered a topological invariant of the system (see Table 2). This means that imprecision in the geometry does not impact the dynamics (propagation speed and phase relationship), which is a rather attractive feature, when SWs are operating as information carriers in 3D networks^41–43^. The absence of any frequency gap at zone boundary is not surprising, since the magnetization is uniformly distributed (full saturation along y-direction), and the geometric discontinuity along x-direction is not creating any magnetic poles.

**Table 2:** Values of the average group velocity (m/s) of the spin wave propagating along either coordinates, for the two magnetic configurations.

Speed (m/s)	${\varvec{M}}\parallel \mathbf{y}$		${\varvec{M}}\parallel \mathbf{x}$			
	BA $\left({\varvec{k}}\parallel \mathbf{M}\right)$	DE $\left({\varvec{k}}\perp \mathbf{M}\right)$	BA $\left({\varvec{k}}\parallel \mathbf{M}\right)$	DE $\left({\varvec{k}}\perp \mathbf{M}\right)$	Edge $\left({\varvec{k}}\parallel \mathbf{M}\right)$	Edge $\left({\varvec{k}}\perp \mathbf{M}\right)$
**Undulation amplitude** ***A*** **(nm)**						
0 nm (film)	−688.3	3171	−679.7	3171	–	–
20 nm	−727.2	3005	−433.4	2062	−178.6	885.6
30 nm	−721.4	2930	−358.6	2261	−324.0	1189
40 nm	−720.0	2854	−214.6	1787	−508.3	1814
60 nm	−712.8	2710	–	–	−637.9	3338

As explained in the article body, the largest value is got by an edge mode, surprisingly. In configuration ${\varvec{M}}\parallel {\varvec{x}}$ no bulk cell function is found. The negative sign in the values indicates the negative slope of the dispersion (BA character).

### Configuration $M\parallel \mathrm{x}$

**Figure 7:**
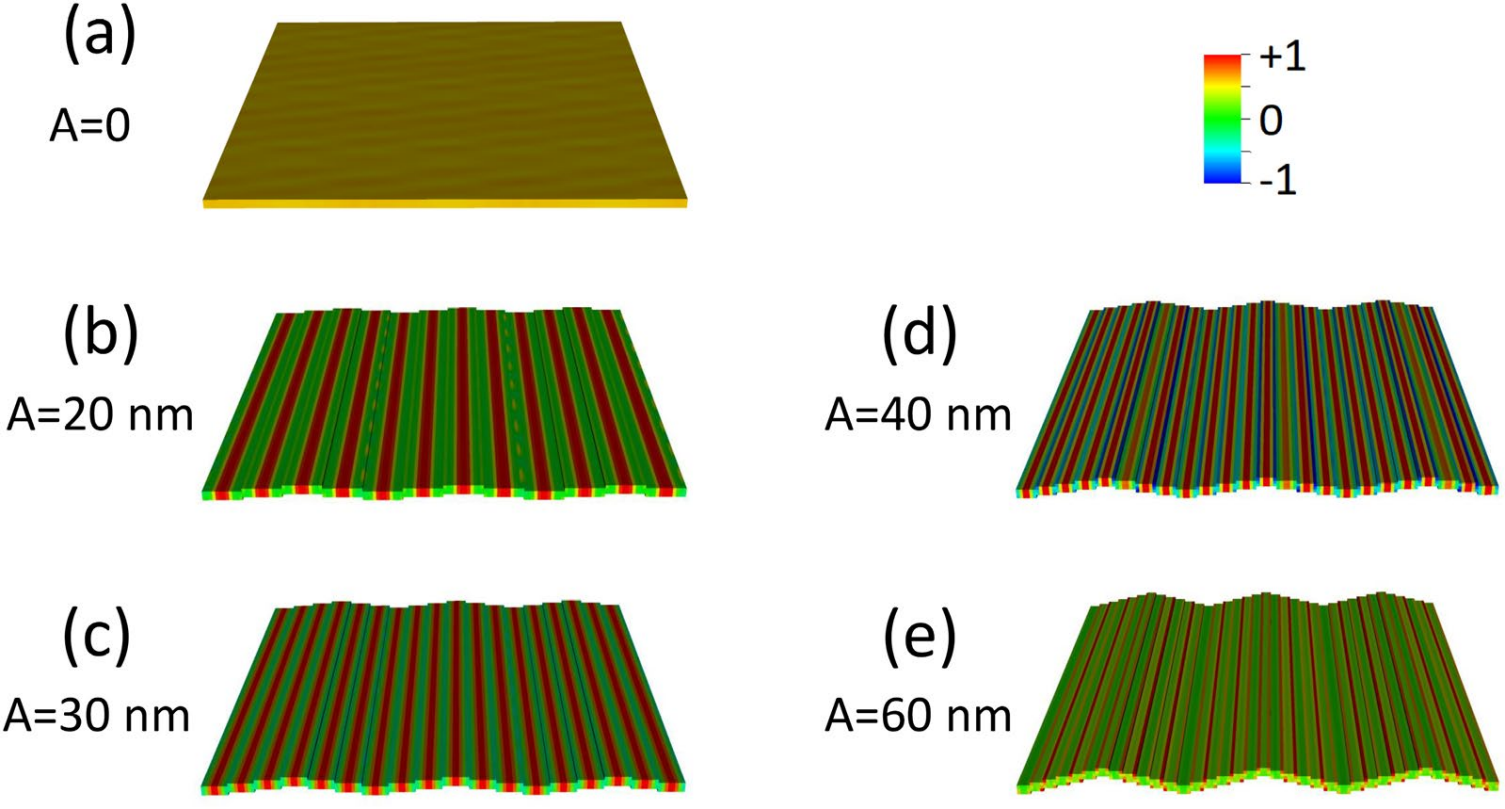
Real part of the out-of-plane (z) dynamic magnetization phase profile (in arbitrary units) for k = 0, in configuration ${\varvec{M}}\parallel {\varvec{x}}$, for increasing undulation amplitude, shown in perspective to highlight the involvement of the third dimension. **(a)** Planar film; **(b)** 4-layer system; **(c)** 6-layer system; **(d)** 8-layer system; **(e)** 12-layer system. The strong dipolar fields, arising because the stripe elements are magnetized along the finite width of the elemental stripes, are at the origin of the localization of the dynamic magnetization at the center of each stripe. In **(e)**, the stripe width is so small (60 nm) that no bulk-like solution is possible even in the single elemental stripe, and the most uniform mode shows nonzero amplitude only at the edges of each elemental stripe: this profile Is qualitatively different from the others. Note that each map is a 3 × 3 repetition of the primitive cell, as an indicative extract of the ideally infinite system.

**Figure 8:**
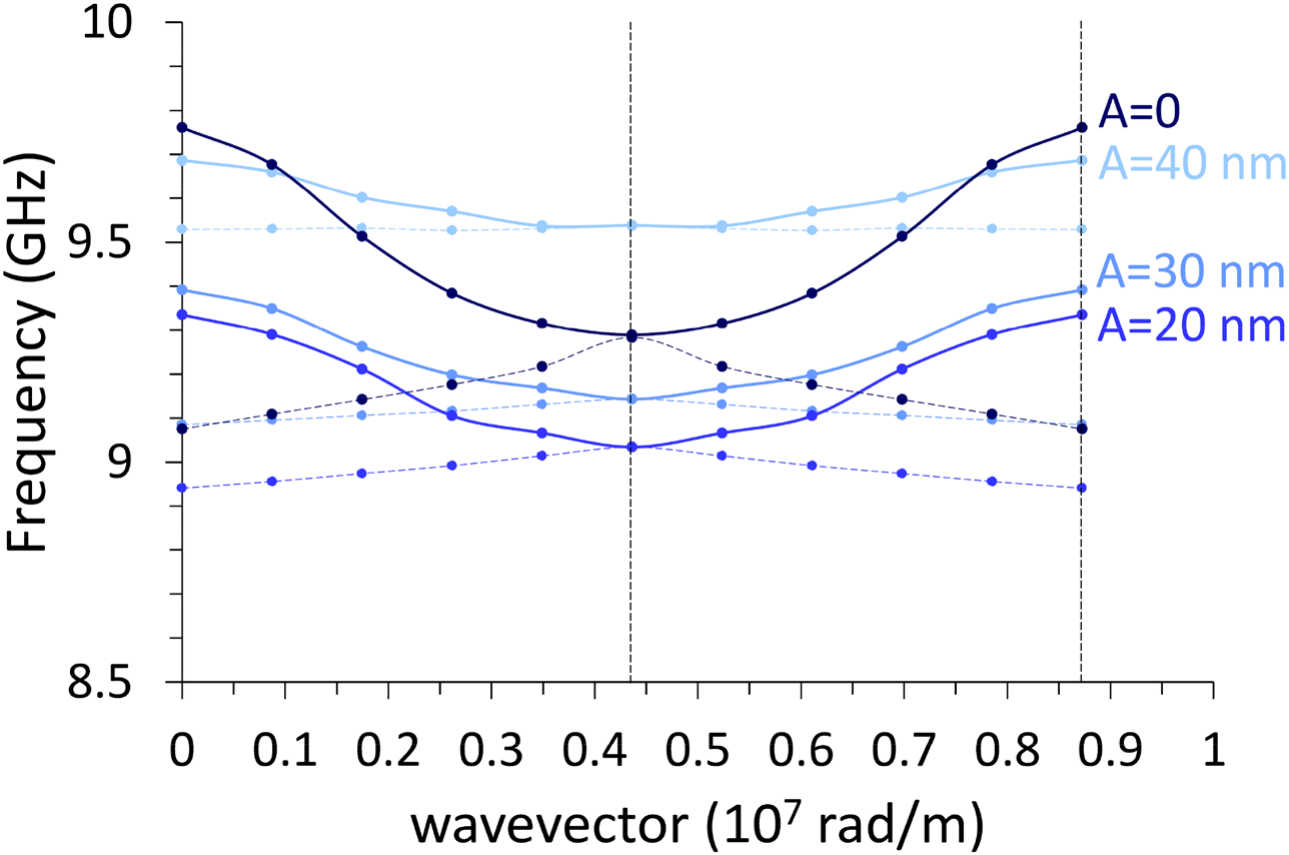
BA mode dispersion curves for the 1st (thick solid lines with full symbols) and mirrored 2nd zone (thin dashed lines) in configuration ${\varvec{M}}\parallel {\varvec{x}}$. The same blue shades from darker to lighter of Fig. 5, correspond to the film (A = 0), and in order, the 4, 6, and 8-stripe system, with indication of the corresponding undulation amplitude A. The 12-stripe (A = 60 nm) system is missing since no bulk solutions are allowed due to the strong dipolar fields arising after magnetizing the stripes along the width.

**Figure 9:**
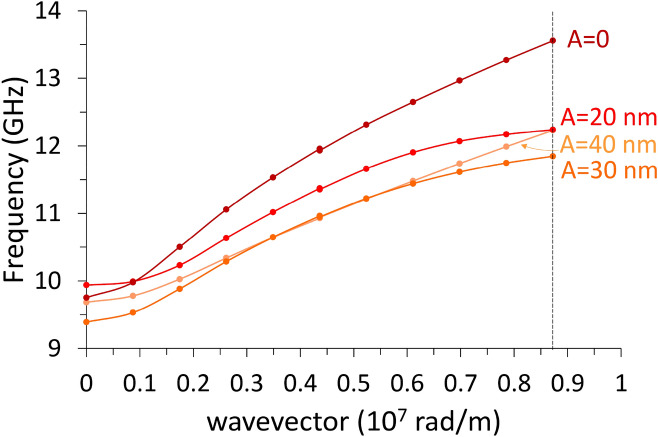
Dispersion curves for the DE mode (${\varvec{k}}\perp {\varvec{M}}$) in the configuration ${\varvec{M}}\parallel {\varvec{x}}$: these waves do not follow the film periodicity, though their frequency varies with the system geometry. Lines are guides for the eyes. Following the same red shades of Fig. 5, the darkest corresponds to the planar film (A = 0), the lighter ones to the 4, 6 and 8-stripe systems, in order. The 12-stripe (A = 60 nm) system is absent since it doesn’t show any bulk solution, but only an edge one, as shown in Fig. 7e.

In this case, when undulation has non-zero amplitude, both magnetization ${\varvec{M}}$ and ${\mathbf{H}}_{\mathrm{eff}}\left(x\right)$ are no more uniform, since the primitive cell is made of elemental stripes each magnetized along the width (hard axis). As it appears in Fig. 2c–e, ${\mathbf{H}}_{\mathrm{eff}}\left(x\right)$ displays pronounced discontinuities and sharp peaks, which separate regions where SW modes can localize; as a consequence, we found the occurrence of two main families of modes, the bulk modes (BM), localized in the center of each stripe, and the edge modes (EM) localized at the boundaries between adjacent stripes (Fig. 7). Now, for ${\varvec{M}}\parallel \mathbf{x}$, the general behavior of the dispersions, independently of the SW wavevector direction and mode character BA (Fig. 8) or DE (Fig. 9) is: from the planar film to the 4-stripe system, when the undulation amplitude $A$ varies from zero to 20 nm, the dispersion experiences a significant frequency drop due to the enhanced dipolar fields, having moved from a uniform to a non-uniform magnetization. Then, for increasing $A$ (and hence, the number of stripes $N$, Table 1) the frequency increases due to the decreasing dipolar energy (discussed above), since adjacent stripes shift far apart. As far as the BA mode is concerned (Fig. 8), this effect is accompanied by a progressive decrease of the mode bandwidth (hence, the group velocity). In fact, the increasing undulation amplitude apparently decreases the coupling among the magnetic moments, since the same amount of magnetic moments is distributed over an increasingly larger arc (the magnetic moment linear density decreases in moving from a straight line to the arc), and this impacts the dynamic fields too, which are responsible for the mode bandwidth.

**Figure 10:**
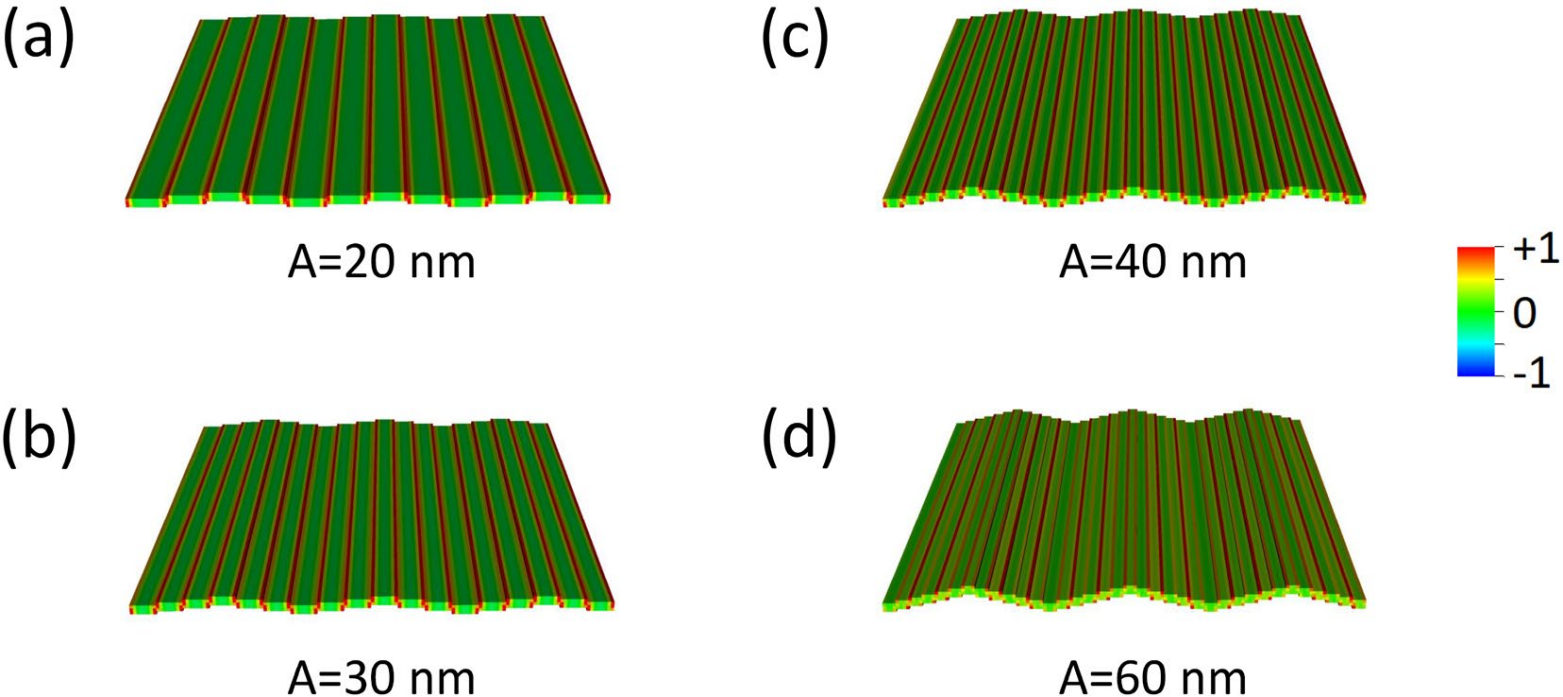
Edge mode phase profiles (real z-components, in arbitrary units) for: **(a)** 4-stripe system; **(b)** 6-stripe system; **(c)** 8-stripe system; **(d)** 12-stripe system (the undulation amplitude A is indicated). Edge mode occur only in the configuration ${\varvec{M}}\parallel {\varvec{x}}$ and only in a non-uniform magnetization (hence, A = 0 is absent). Note that mode **(b)** is taken from Fig. 7e. Each map is a 3 × 3 repetition of the primitive cell, as an indicative extract of the ideally infinite system.

An additional effect arises here: no bulk uniform mode can exist below a given critical stripe width. Indeed, since each stripe is magnetized along the narrow width, the strong dipolar (demagnetizing) fields prevent any coherent spin oscillation involving the bulk of each stripe. For this reason, no bulk solution is found for the 12-stripe system. In this case, only edge solutions exist (see either Fig. 7e or Fig. 10d, which are the same), as found in the literature for similar or analogue systems, with a strongly varying ${\mathbf{H}}_{\mathrm{eff}}\left(x\right)$^44–48^.

### Comments about the occurrence of a frequency gap

It is worth to recall how the opening of a gap at zone boundary comes as a result of two conceptually independent effects. The first one is the linear superposition of two propagating plane waves (with  = 2a, i.e., k = ± π/a), incident and reflected by the medium discontinuities, which interfere because of periodicity and combine into two stationary waves with a quarter of wavelength path difference. The second one relies on the medium physical properties, which must be differently experienced by the two mentioned stationary waves: otherwise, the two waves are indistinguishable with respect to the medium and degenerate in frequency: in order this could occur, the periodic cell must be such that its center has different properties from the edges. While it is clear that a uniform magnetization (configuration ${\varvec{M}}\parallel \mathbf{y}$), despite distributed in a curved geometry, cannot offer such symmetry breaking, it is less trivial when ${\varvec{M}}\parallel \mathbf{x}$ and ${\varvec{k}}\parallel \mathbf{x}$ (BA mode), and must be based on the specific magnetization profile. In a recent paper, this effect was already seen but not explained^49^. Details will be given in the “Discussion” section below.

As far as the DE mode is concerned (Fig. 9), we found a similar frequency drop, and a similar SW bandwidth reduction, moving from the planar film to the 4-stripe system, i.e., varying $A$ from zero to 20 nm. In addition, an apparent, remarkable effect is that the dispersion for the 8-stripe system ($A=$ 40 nm) seems intermediate between the 4 and the 6-stripe systems, depending on the wavevector. This effect is here just spotted, but would require wider studies to be fully understood. Nevertheless, we observe how the frequency variations among the three systems (4, 6, and 8-stripe systems) are in any case very small in this configuration, around or below 0.5 GHz, so that the frequency variations due to different undulation amplitudes, at a specific k, is just a matter of computational error, or, in possible measurements, experimental resolution.

**Figure 11:**
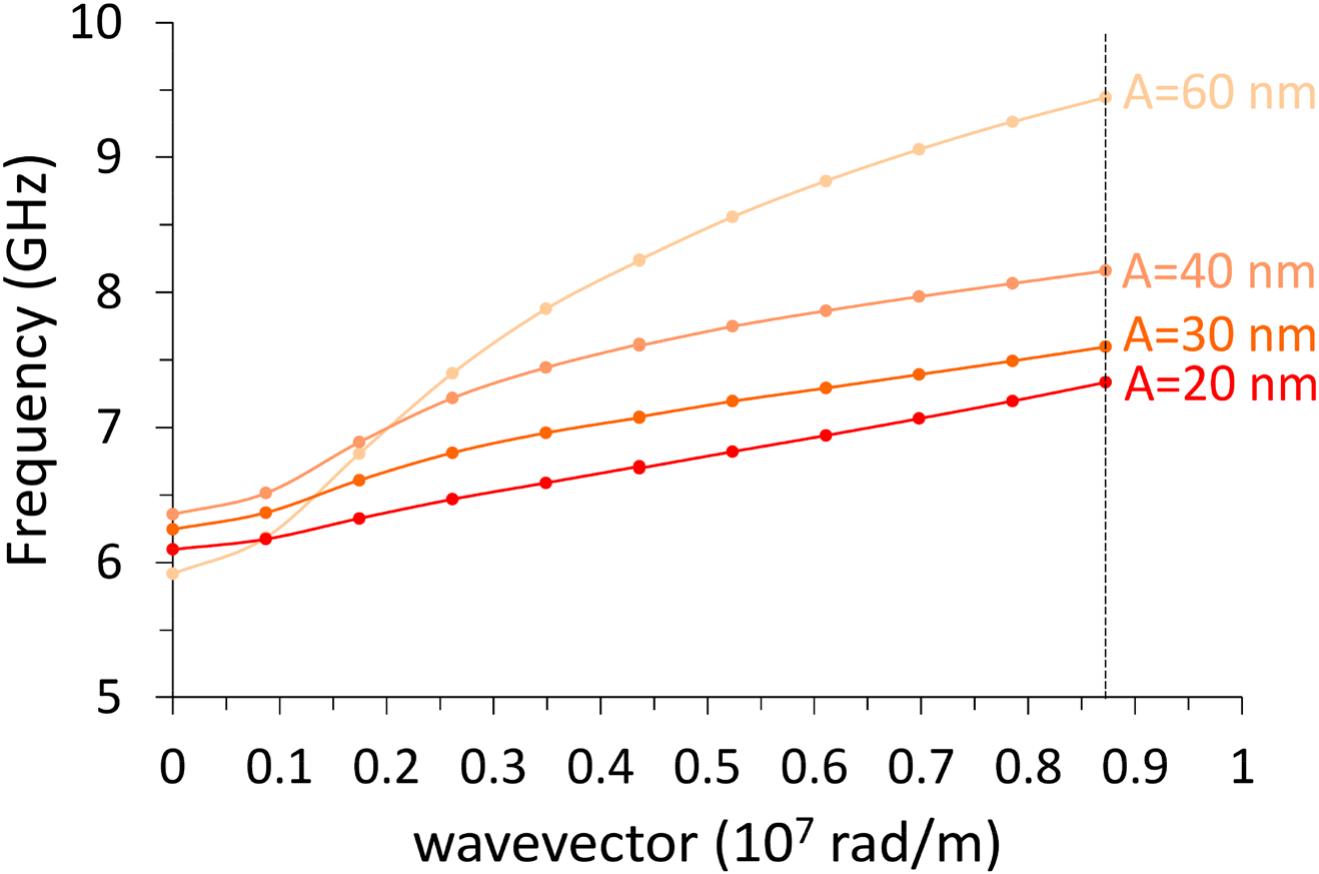
Dispersion relations of the edge mode with ${\varvec{k}}\perp {\varvec{M}}$, in a film with progressive undulation amplitude A, from A = 20 nm (4-stripe system) to A = 60 nm (12-stripe system), indicated by the same red shades, from the darker to lighter, of Fig. 5. No periodicity is expected along the propagation direction ($y$-axis): the vertical dashed line marks the limit wavevector in the calculations. The first three systems show a progressive frequency increase, but note how the 12-stripe edge dispersion (in black) qualitatively differs from the others, due to the strongest demagnetizing fields.

**Figure 12:**
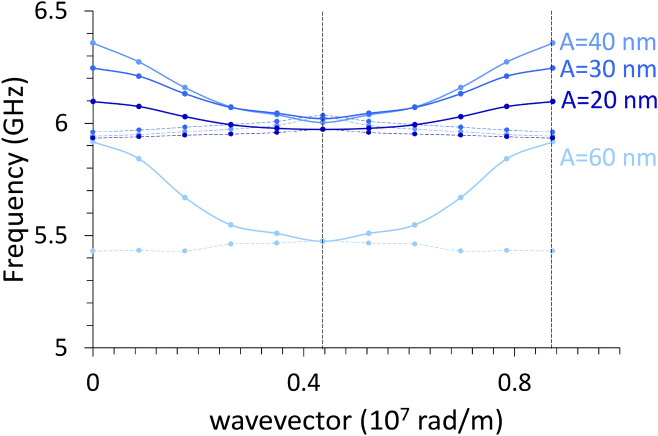
Dispersion relations of the edge mode with ${\varvec{k}}\parallel {\varvec{M}}$ up to the second zone boundary, in a film with progressive undulation amplitude A, from 20 nm (4-stripe system, the darkest blue line) to 60 nm (12-stripe system, the lightest blue line), indicated by the blue shades from darker to lighter. Continuous lines refer to the first Brillouin zone, dashed lines to the (mirrored) second zone. The first three systems show a progressive frequency increase, while the 12-stripe edge dispersion (A = 60 nm) is at a very lowest frequency. The frequency band corresponding to the first zone is progressively increasing with the undulation amplitude.

Finally, we discuss the dispersion curves for the uniform edge mode (profile shown in Fig. 10) either when ${\varvec{k}}\parallel \mathbf{y}$ (Fig. 11, showing no periodicity) or ${\varvec{k}}\parallel \mathbf{x}$ (Fig. 12, showing periodicity since oscillation is along the undulation direction). The edge mode is a Bloch spin wave with the cell function showing significant amplitude only at the elemental stripe edges. Edge modes can exist only because of a non-uniform ${\mathbf{H}}_{\mathrm{eff}}\left(x\right)$, hence only when the film is not a straight plane. We see how the increase in the undulation amplitude causes a frequency increase for all 4, 6, and 8-stripe system, but not for the 12-stripe system, which shows a different behavior in both cases. This diversity occurs because the width of the elemental stripe is too small^45^(i.e., w = 60 nm) and the corresponding dipolar fields are very stronger. For this reason, in both Figures the curve relevant to the 12-stripe system departs from the others. Remarkably, the 12-stripe system shows a significant bandwidth (independently of the propagation direction), which is a consequence of the large dynamic stray fields caused by the dynamic magnetization.

## Discussion

**Figure 13:**
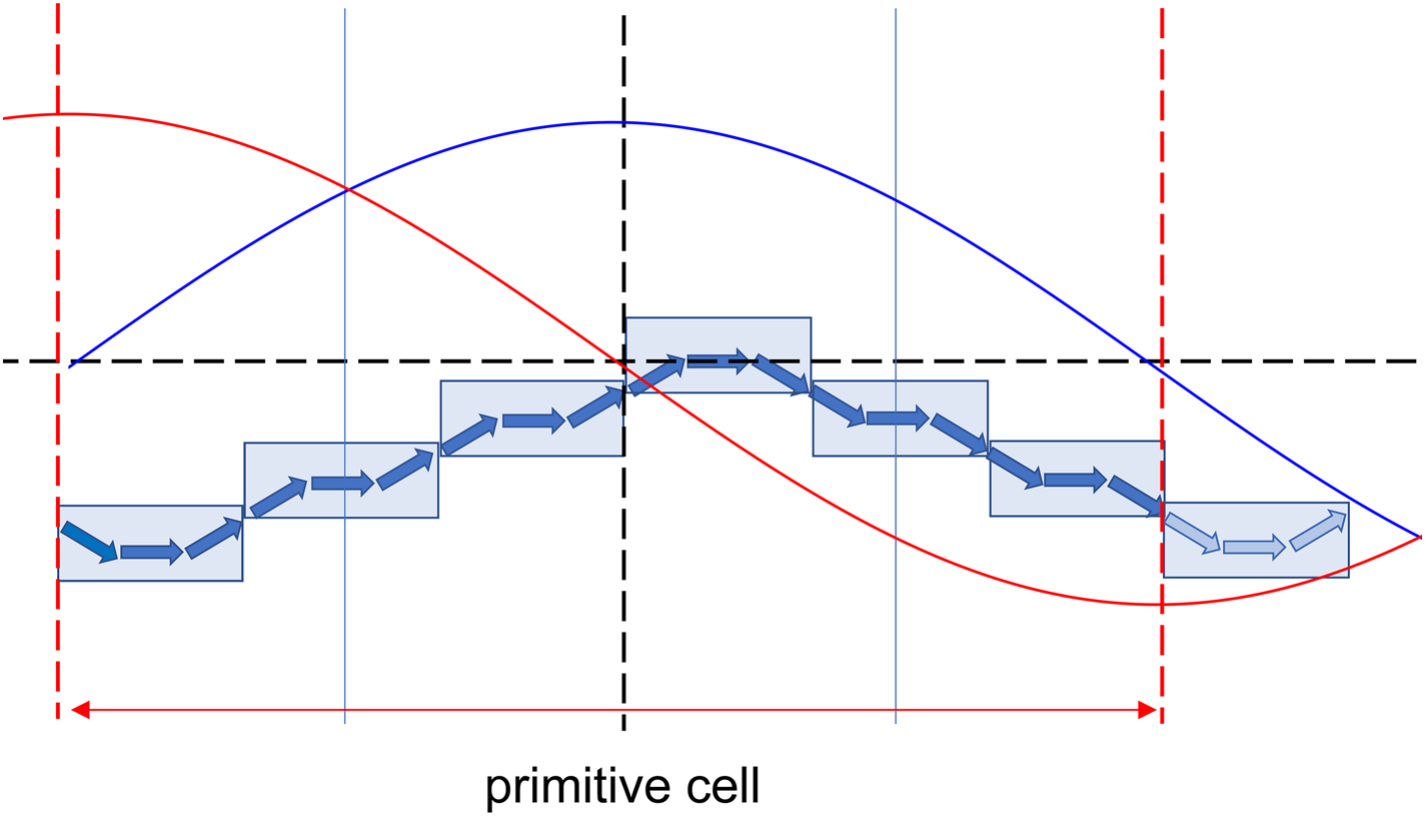
Sketch of the correspondence between magnetization undulation and spin oscillation wavelength at zone boundary (k = π/a) for the system made of 6-stripes. Black dashed lines set the reference system, the blue and red lines represent the two diffracted waves at zone boundary, having a phase difference of π/2. Apparently, even when M is not uniform, the two solutions experience point by point exactly the same magnetization flux lines (illustrated by the blue arrows), resulting indistinguishable and hence in principle degenerate in frequency. This happens any time that λ/4 is an integer of the stripe width w.

As a consequence of the specific representation we chose for the film undulation, we have the following results. Firstly, the spin wave periodicity is observed only along the undulation direction, expressly on the DE mode when the film is magnetized perpendicular to the undulation direction (*y*-axis), and the BA mode when the film is magnetized parallel to the undulation direction (*x*-axis). Moreover, the variation in frequency is experienced by spin waves independently of the propagation direction or the magnetization configurations, because the geometric changes influence the whole system energetics. In other words, the undulation changes vary the whole system total energy, which in turn can make the system either more stable (stiffer), determining a general frequency increase, or less stable (softer), determining a general frequency decrease^50,51^. Interestingly, due to the specific symmetry of the primitive cell that we chose, we do not have any frequency gap across the first and second Brillouin zone for any propagating spin wave. The two wavefunctions, forming at zone boundary ($\lambda =2a$) as the consequence of diffraction, happen to have both node and maxima at the edges across two adjacent stripes, and independently of its direction (*x* or *y*) the magnetization shows the same flux line orientations in each stripe along the primitive cell (see Fig. 13), so that a shift of λ/4 would not change how a solution experiences the energetics of the system: hence, they happen to be degenerate in frequency in principle. Since the magnetization distribution along x direction is the same independently of the position of the elemental stripe in the primitive cell, in general, Bragg diffraction occurs when

$$k=\frac{\pi }{w}=\frac{N\pi }{a}$$

and in our case N (number of elemental stripes in a primitive cell) is always even, so we would not see any mode repulsion at any zone boundary, i.e., at any $\frac{N}{2}\frac{\pi }{a}$.

In this class of problems, i.e., undulated films, the absence of any mode repulsion at zone boundary is independent of the elemental stripe size *w* though which it is constructed, and, in the limit w → 0 (smooth continuous film), mode repulsion must be still absent and hence the forbidden gap cannot be found at any k, despite periodicity, as a very general feature. Periodicity is in fact a necessary but not sufficient condition for a forbidden gap formation, the other necessary condition is either discontinuity in any physical parameter, say $f$, across the primitive cell, typically its left limit differing from the right limit $f\left({a}^{-}\right)\ne f\left({a}^{+}\right)$ (as in 1-D lattice of stripes of different materials), or non-uniformity in case of continuity of $f$, e.g. $f\left(\frac{a}{2}\right)\ne f\left(0\right)$, i.e., the value of $f$ at the center of the primitive cell must be different from that at the cell boundaries (as either in nanomagnet or antidot lattices). In this way, since the energetics is ruled by $f$, the two diffracted waves with an optical path length difference $\delta =\frac{\lambda }{4}\equiv \frac{a}{2}$ would both have $\lambda =2a$, which corresponds to zone boundary ($k=\frac{\pi }{a}$), but would experience differently the physical parameter $f$, getting a different frequency. In our problems, $f$ is the magnetization function $M\left(x\right)$, and, importantly, due to the chosen geometry, not only it is always continuous (i.e., $M\left({a}^{-}\right)=M\left({a}^{+}\right)$), but furthermore, the rule $f\left(\frac{a}{2}\right)=f\left(0\right)$, i.e., in our case, $M\left(\frac{a}{2}\right)=M\left(0\right)$, holds even when the undulated film is magnetized along the undulation direction, with a non-uniform magnetization. Besides, as already remarked, both diffracted waves experience the same magnetization flux lines, even if at different points in the primitive cell. Hence, we can’t expect any forbidden gap in any case in principle.

### Edge modes

Going a little further, we discuss a few other remarkable features. Despite localized in narrow regions, the edge modes in configuration ${\varvec{M}}\parallel \mathbf{x}$ show appreciable frequency bandwidth, in particular it increases on increasing undulation amplitude. Usually localization decreases the dynamic coupling fields, but in our geometry, the number of edges increases with the undulation amplitude (i.e., the number of stripes) and hence, the more the edges between adjacent stripes, the larger the dynamic coupling fields and hence the mode bandwidth. Their appreciable average group velocity allows to look to these modes as information carriers too, as done for their bulk counterparts. We computed the average group velocities of all the SW modes in either configurations ${\varvec{M}}\parallel \mathbf{y}$ and ${\varvec{M}}\parallel \mathbf{x}$, collected in Table 2. While, as expected, the fastest bulk wave is a DE mode in the planar film, surprisingly the absolute fastest spin wave is an edge mode for the maximum undulation amplitude in configuration ${\varvec{M}}\parallel \mathbf{x}$. In ordinary conditions, spin wave localization fits with low frequencies as a consequence of high dipolar fields^52^: but in our geometry, due to the large number of stripes forming the primitive cell (in the 12-stripe system), and magnetized along the hard axis, there is a summation effect which enhances the dynamic stray fields. In a sense, limiting to our results, we might say in a catch phrase that undulation accelerates a special class of spin waves, the backward edge mode.

**Figure 14:**
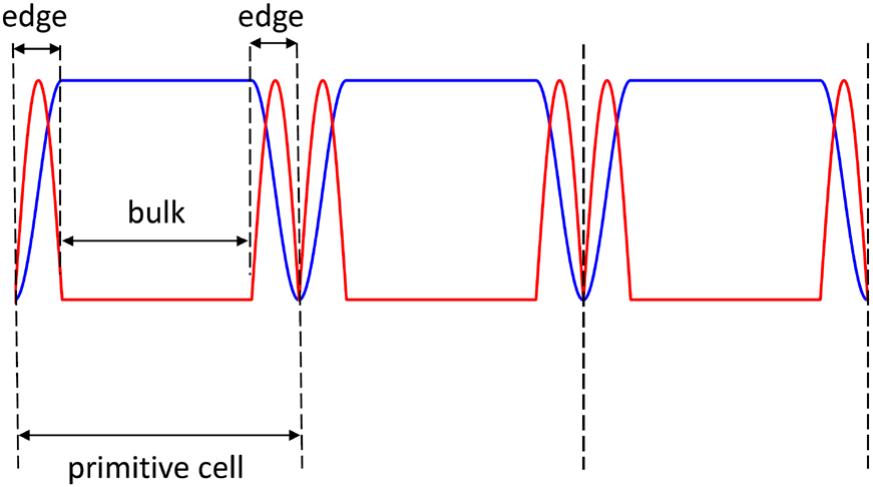
Illustrative sketch of a possible continuous (w → 0) periodic undulation of the magnetization ${M}_{x}\left(x\right)$ (blue line) superposed to the corresponding divergence (absolute value, red line), showing the occurrence of distinct bulk/edge regions (indicatively) for localization of spin wave amplitude within the cell wavefunction. The peaks of the divergence correspond to the minima of the internal effective field.

The reader might observe that any real sample, in configuration ${\varvec{M}}\parallel \mathbf{x}$, would not have the strong discontinuities we found that are associated to the serrated shape of our stripe-based representation: the real magnetization should in fact vary smoothly. We reply noticing that nevertheless a divergence would display peaks at each change of curvature of the magnetic undulation (specifically, the component *M*_*x*_), signaling the presence of magnetic poles and effective walls for spin waves, dividing the primitive cell into bulk and edge regions (Fig. 14). Hence, even in a smoothly varying geometry, undulated films can host edge modes as well, similarly to what found in our calculations. The difference is that only two edge regions per primitive cell can appear, while in our discretized case we had two edge regions per stripe. Besides, a device actually built of stripes might be of interest in the context of a dual-band signal delivery: as apparent by our calculations, edge mode frequencies are below 8 GHz, while bulk BA/DE modes are beyond 9 GHz, so there is a gap of more than 1 GHz, and the excitation areas are complementary (bulk/edges) and the related SW intensity is considerable: hence the perspective of simultaneous delivery of two signals is conceivable in appropriate devices inspired by our composite structure, as suggested in the introductory paper ref.^53^.

Finally, to broaden the generality of our results, we suggest that the continuous magnetization undulation could be attained not only by a geometric deformation of the planar film but also through any appropriate nanomagnet lattice layers on a planar film, or even through a multiferroic multilayer that, via magnetoelastic coupling, can provide a periodic effective undulation amplitude of the magnetic anisotropy, and hence magnetization, which can be tuned on demand by any external electric fields (electrically driven magnonics).

In conclusion, we investigated how a geometric undulation in a continuous film modifies the phase profile and dispersion relation for the SW not only propagating along the undulation direction, but also perpendicular to it. We studied the cases when the film is magnetized parallel or perpendicular to the undulation direction, i.e., when the magnetization is uniform or non-uniform, and provide rules for the occurrence (or absence) of a forbidden gap at zone boundary. We discussed the coexistence of periodic waves together with non-periodic waves, and, on the other side, of bulk waves together with edge waves when the magnetization is not uniform. We addressed the conditions at which the SW group velocity becomes topologically invariant against undulation alterations. We found how special edge modes, forming in such a 3D structure, show a large frequency bandwidth comparable to or even larger than the bulk wave ones, hence allowing an additional channel for simultaneous information delivery.

## Methods

We used the object oriented micromagnetic framework (OOMMF) simulator^54^, with implemented periodic boundary conditions, to compute the distribution of the equilibrium magnetization for each system, as well as the internal effective field profiles. The primitive cell, a square with side 720 nm and a number of 10-nm-thick layers depending on the overall height ${d}_{N}^{MAX}$ (Table 1), was discretized through micromagnetic cells with dimensions 5 × 5x10 nm^3^ and represented by a series of adjacent, 30-nm thick stripes following the geometry shown in Fig. 1. The magnetic parameters were typical for permalloy material: exchange stiffness constant $A=10.0 pJ/m$, saturation magnetization ${M}_{S}=800 kA/m$ and the gyromagnetic ratio $\gamma =185 \mathrm{rad GHz}/\mathrm{T}$. The magnetization maps were the input of the DMM software^55,56,59^, which computes the frequency and phase profiles of the dynamic magnetization both at any given applied field and wavevector magnitude and direction. In our case the applied field was ${\mu }_{0}{H}_{0}=0.1 \mathrm{T}$, while the wavevector magnitude *k* was taken at discrete points, from 0 to 0.5 with steps of 0.1 in units of the Brillouin zone width, and then converted into absolute values rad/m.

### Outline of the DMM

**Figure 15:**
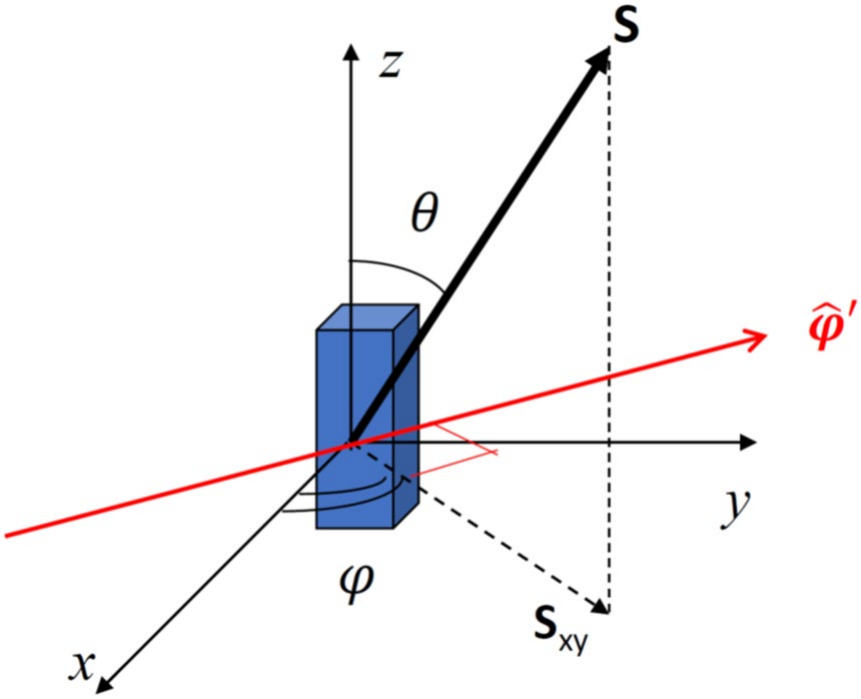
Reference system showing the coordinates of the precession of a spin around z-axis (azimuthal angle variation) or around $\widehat{\mathbf{\varphi }}\mathrm{^{\prime}}$ (in red), i.e., the azimuthal axis orthogonal to the plane formed by z-axis and **S** (polar angle variation).

As discussed at the beginning of the Results Section, we divide the primitive cell into a mesh of micromagnetic cells (prisms), inside which magnetization is uniform: this assumption is justified by the small size of these micromagnetic cells, comparable to the exchange length. Hence, our problem consists both of many micromagnetic cells interacting with each other inside the primitive cell, and also many primitive cells (at different lattice points) interacting with each other. Each micromagnetic cell, across the mesh, has a fixed magnetic moment, which precesses around its local equilibrium direction, identified by the equilibrium azimuthal and polar angles $$\varphi ,\theta$$ (found through OOMMF). The equilibrium direction in a specific cell is determined by the effective field generated by all the other magnetic moments (through dipolar and exchange interactions) and the applied external field (if present). In the precession motion of a single spin $${\varvec{S}}$$, the generalized coordinates $${q}_{n}$$ are the small variations both of azimuthal $$({q}_{1}=\delta \varphi )$$ and polar $$\left({q}_{2}=\delta \theta \right)$$ angles around the corresponding rotation axis, and the conjugate momenta $$({p}_{1}$$ and $${p}_{2})$$ are the projection of the related spin variation $$\delta {\varvec{S}}$$ to the corresponding rotation axis ($${\widehat{{\varvec{e}}}}_{rotation}$$)^57,58^:

$$\left\{\begin{array}{l}{q}_{n}=\delta \varphi ,\delta \theta \\ {p}_{n}=\delta {\varvec{S}}\cdot {\widehat{{\varvec{e}}}}_{rotation}\end{array}\right.$$

with $n=\mathrm{1,2}$. The precessing spin ${\varvec{S}}$ is linked to the magnetization $\mathbf{M}={M}_{s}\widehat{\mathbf{m}}$ through the following rule:

$$\mathbf{S}=\frac{{\varvec{\upmu}}}{\gamma }=\frac{{M}_{s}V\widehat{\mathbf{m}}}{\gamma }=\frac{{M}_{s}V}{\gamma }\left(\begin{array}{c}\mathrm{sin}\theta \mathrm{cos}\varphi \\ \mathrm{sin}\theta \mathrm{sin}\varphi \\ \mathrm{cos}\theta \end{array}\right)$$

where ${\varvec{\upmu}}$ is the magnetic moment of a single elemental prism (micromagnetic cell), *V* is the volume of the prism, $\gamma$ is the gyromagnetic ratio, ${M}_{s}$ is the saturation magnetization of the material, and the unit vector $\widehat{\mathbf{m}}$ is expressed in spherical coordinates. With this relation, the generalized coordinates and conjugate momenta can be written as (Fig. 15):

$$\left\{\begin{array}{l}{q}_{1}=\delta \varphi \\ {p}_{1}=\delta {\varvec{S}}\cdot {\widehat{{\varvec{e}}}}_{z}\equiv \delta {S}_{z}=\frac{v{M}_{s}}{\gamma }\delta {m}_{z}=\frac{v{M}_{s}}{\gamma }\left(-\mathit{sin}\theta \delta \theta \right)\end{array}\right.$$

$$\left\{\begin{array}{l}{q}_{2}=\delta \theta \\ {p}_{2}=\delta {\varvec{S}}\cdot \widehat{\boldsymbol{\varphi }}{^{\prime}}\equiv \delta {S}_{xy}={S}_{xy}\delta \varphi =\left(\left|S\right|\mathit{sin}\theta \right)\delta \varphi =\frac{v{M}_{s}}{\gamma }\mathit{sin}\theta \delta \varphi \end{array}\right.$$

Then, the Hamilton equations, relevant for a single micromagnetic cell (i.e., a single magnetic moment) read:

$$\left\{\begin{array}{l}\frac{\partial {q}_{\mathrm{1,2}}}{\partial t}=\frac{\partial H}{\partial {p}_{\mathrm{1,2}}}\\ \frac{\partial {p}_{\mathrm{1,2}}}{\partial t}=-\frac{\partial H}{\partial {q}_{\mathrm{1,2}}}\end{array}\right.$$

where H is the Hamiltonian of the system, i.e., the energy function including Zeeman, exchange and dipolar interactions (for the micromagnetic representation of these interactions see refs.^55,59^). After substitution we get:

$$\left\{\begin{array}{l}\frac{\partial \delta \varphi }{\partial t}=-\frac{\gamma }{V{M}_{s}\mathit{sin}\theta }\frac{\partial H}{\partial \delta \theta }\\ \frac{\partial \delta \theta }{\partial t}=+\frac{\gamma }{V{M}_{s}\mathit{sin}\theta }\frac{\partial H}{\partial \delta \varphi }\end{array}\right.$$

Now, we have to consider that the Hamiltonian includes interactions between a given micromagnetic cell and all the (for dipolar) or some (for exchange) others. Hence it is necessary to index the cells: we address a given reference micromagnetic cell with index *k*, while all the others, interacting with that, will be indexed with *j*. The total number of cells in the micromagnetic mesh is N (maximum value of the indices, and, hence, system size).

Furthermore, we exploit the fact that, for harmonic precession, we also have:

$$\left\{\begin{array}{c}\delta {\varphi }_{k}=\delta {\varphi }_{k}^{0}{e}^{i\omega t}\\ \delta {\theta }_{k}=\delta {\theta }_{k}^{0}{e}^{i\omega t}\end{array}\right.$$

$$\left\{\begin{array}{c}\frac{\partial \delta {\varphi }_{k}}{\partial t}=i\omega \delta {\varphi }_{k}\\ \frac{\partial \delta {\theta }_{k}}{\partial t}=i\omega \delta {\theta }_{k}\end{array}\right.$$

where $\delta {\varphi }_{k}^{0}$ and $\delta {\theta }_{k}^{0}$ are the oscillation amplitudes and $\omega /2\pi$ the frequency. Then, we introduce the energy density $U=H/V$, and, since a harmonic behavior involves small oscillations, we expand it in Taylor series up to second order. Considering that we deal with equilibrium systems (found by OOMMF) we retain only the quadratic term, and then, with just a simple algebraic manipulation, we get a linear system, describing the precession of the magnetization in the *k*th-cell, which is interacting with all the other *j*th-cells of the system:

4 $$\left(\begin{array}{cc}-\frac{1}{\mathit{sin}{\theta }_{k}}{\sum }_{j}\frac{{\partial }^{2}U}{\partial \delta {\theta }_{k}\partial \delta {\varphi }_{j}}\partial \delta {\varphi }_{j}& -\frac{1}{\mathit{sin}{\theta }_{k}}{\sum }_{j}\frac{{\partial }^{2}U}{\partial \delta {\theta }_{k}\partial \delta {\theta }_{j}}\partial \delta {\theta }_{j}\\ \frac{1}{\mathit{sin}{\theta }_{k}}{\sum }_{j}\frac{{\partial }^{2}U}{\partial \delta {\varphi }_{k}\partial \delta {\varphi }_{j}}\partial \delta {\varphi }_{j}& \frac{1}{\mathit{sin}{\theta }_{k}}{\sum }_{j}\frac{{\partial }^{2}U}{\partial \delta {\varphi }_{k}\partial \delta {\theta }_{j}}\partial \delta {\theta }_{j}\end{array}\right)-i\omega \frac{{M}_{s}}{\gamma }\left(\begin{array}{c}\delta {\varphi }_{k}\\ \delta {\theta }_{k}\end{array}\right)=0$$

This linear system, after appropriate exchanges of rows (or columns), is cast into an eigenvalue/eigenvector problem $$\left(\overset{\lower0.5em\hbox{$\smash{\scriptscriptstyle\frown}$}}{{\varvec{B}}}-{\lambda }_{k}\overset{\lower0.5em\hbox{$\smash{\scriptscriptstyle\frown}$}}{{\varvec{I}}}\right){{\varvec{v}}}_{k}=0$$, namely:

$$\left(\begin{array}{ccc}\begin{array}{cc}{B}_{11}& {B}_{12}\\ {B}_{21}& {B}_{22}\end{array}& \cdots & \cdots \\ \vdots & \ddots & \\ \vdots & & \ddots \end{array}\right)\left(\begin{array}{c}\begin{array}{c}\delta {\varphi }_{1}\\ \delta {\theta }_{1}\end{array}\\ \begin{array}{c}\delta {\varphi }_{2}\\ \delta {\theta }_{2}\end{array}\\ \vdots \\ \begin{array}{c}\delta {\varphi }_{k}\\ \delta {\theta }_{k}\end{array}\\ \vdots \end{array}\right)-{\lambda }_{k}\left(\begin{array}{c}\begin{array}{c}\delta {\varphi }_{1}\\ \delta {\theta }_{1}\end{array}\\ \begin{array}{c}\delta {\varphi }_{2}\\ \delta {\theta }_{2}\end{array}\\ \vdots \\ \begin{array}{c}\delta {\varphi }_{k}\\ \delta {\theta }_{k}\end{array}\\ \vdots \end{array}\right)=0$$

(*k* index goes from 1 to N) which provides non-trivial solutions only if $$\mathit{det}\left(\overset{\lower0.5em\hbox{$\smash{\scriptscriptstyle\frown}$}}{{\varvec{B}}}-{\lambda }_{k}\overset{\lower0.5em\hbox{$\smash{\scriptscriptstyle\frown}$}}{{\varvec{I}}}\right)=0$$. We address $$\overset{\lower0.5em\hbox{$\smash{\scriptscriptstyle\frown}$}}{{\varvec{B}}}$$ as the *dynamical matrix*: note how its N^2^ elements correspond to the restoring torque acting on the magnetic moment of each micromagnetic cell. From the resulting characteristic polynomial it is possible to get the eigenvalues:

$${\lambda }_{k}=i{\omega }_{k}\frac{{M}_{s}}{\gamma }$$

which can be used to get the SW frequencies ${\omega }_{k}/2\pi$, and the eigenvectors:

$${{\varvec{v}}}_{k}=\left(\begin{array}{c}\begin{array}{c}\delta {\varphi }_{1}\\ \delta {\theta }_{1}\end{array}\\ \begin{array}{c}\delta {\varphi }_{2}\\ \delta {\theta }_{2}\end{array}\\ \vdots \\ \begin{array}{c}\delta {\varphi }_{k}\\ \delta {\theta }_{k}\end{array}\\ \vdots \end{array}\right)$$

which represent the precession angles of the magnetization in each micromagnetic cell and can be transformed into the full SW mode spatial profiles considering that *k* goes from 1 to N. Note that the extension of the ferromagnetic body along the three cartesian coordinates $x,y,z$ is just a matter of indexing, so that position $\mathbf{r}$ of any micromagnetic cell inside the three-dimensional mesh can be seen as $\mathbf{r}\left(x,y,z\right)\equiv {\mathbf{r}}_{k}$. Clearly, the system size N determines also the number N of possible normal modes ${({\varvec{v}}}_{k})$.

When dealing with periodic systems, we have a set of lattice points at position $\mathbf{R}$ (any linear combination of the primitive vectors). Note that our systems involve all the three dimensions, but periodicity is only along $x,y$. At each lattice point $\mathbf{R}$ we have exactly the same magnetization distribution that we have in the primitive cell at $\mathbf{R}=0$ (and the same index *k* to address all the micromagnetic cells at position ${\mathbf{r}}_{k}$ for any $\mathbf{R}\ne 0$). The linear system above [Eq. (4)], in this new case, corresponds to the dynamics within a single primitive periodic cell only, and hence must be extended to include the interactions of this primitive cell (usually taken, as a reference, at $\mathbf{R}=0$) with all the other periodic cells at other lattice points $\mathbf{R}\boldsymbol{^{\prime}}$ (introducing a summation over $\mathbf{R}\boldsymbol{^{\prime}}$). Since the magnetization has the same distribution $\mathbf{M}\left(x,y,z\right)$ in any periodic cell, exploiting the Bloch theorem and introducing the wavevector **K**, this can be easily shown to give an extra factor (i.e., ${e}^{i\mathbf{K}\cdot {\mathbf{R}}^{\boldsymbol{^{\prime}}}}$) in Eq. (4), and the dynamic equation for periodic systems is transformed into:

$$\left(\begin{array}{cc}-\frac{1}{\mathit{sin}{\theta }_{k}}{\sum }_{j, {\mathbf{R}}^{\boldsymbol{^{\prime}}}}\frac{{\partial }^{2}U}{\partial \delta {\theta }_{k}\partial \delta {\varphi }_{j{\mathbf{R}}^{\boldsymbol{^{\prime}}}}}{e}^{i\mathbf{K}\cdot {\mathbf{R}}^{\boldsymbol{^{\prime}}}}\partial \delta {\varphi }_{j}& -\frac{1}{\mathit{sin}{\theta }_{k}}{\sum }_{j, {\mathbf{R}}^{\boldsymbol{^{\prime}}}}\frac{{\partial }^{2}U}{\partial \delta {\theta }_{k}\partial \delta {\theta }_{j}}{e}^{i\mathbf{K}\cdot {\mathbf{R}}^{\boldsymbol{^{\prime}}}}\partial \delta {\theta }_{j}\\ \frac{1}{\mathit{sin}{\theta }_{k}}{\sum }_{j, {\mathbf{R}}^{\boldsymbol{^{\prime}}}}\frac{{\partial }^{2}U}{\partial \delta {\varphi }_{k}\partial \delta {\varphi }_{j{\mathbf{R}}^{\boldsymbol{^{\prime}}}}}{e}^{i\mathbf{K}\cdot {\mathbf{R}}^{\boldsymbol{^{\prime}}}}\partial \delta {\varphi }_{j}& \frac{1}{\mathit{sin}{\theta }_{k}}{\sum }_{j, {\mathbf{R}}^{\boldsymbol{^{\prime}}}}\frac{{\partial }^{2}U}{\partial \delta {\varphi }_{k}\partial \delta {\theta }_{j{\mathbf{R}}^{\boldsymbol{^{\prime}}}}}{e}^{i\mathbf{K}\cdot {\mathbf{R}}^{\boldsymbol{^{\prime}}}}\partial \delta {\theta }_{j}\end{array}\right)-i{\omega }_{k}\frac{{M}_{s}}{\gamma }\left(\begin{array}{c}\delta {\varphi }_{k}\\ \delta {\theta }_{k}\end{array}\right)=0$$

Here, index *k* runs for the micromagnetic cells inside the reference primitive cell ($\mathbf{R}=0$), while index *j* runs for the micromagnetic cells in any primitive cell at lattice point ${\mathbf{R}}^{\boldsymbol{^{\prime}}}$ (including ${\mathbf{R}}^{\boldsymbol{^{\prime}}}=0$). Even in this case, after an appropriate change of rows/columns, the linear system can be cast into an eigenvalue/eigenvector problem, which has exactly the same system size N of the previous one Eq. (4), thanks to the translational symmetry and the Bloch theorem. This time, we have an additional parameter, which is the wavevector **K** at which the calculation is performed, which must be provided as an input, and in general can be any arbitrary point in the reciprocal space. Hence, the solutions (eigenvectors) must be intended as Bloch waves following Eq. (1), which (with the present notation) reads:

$$\delta {m}_{\mathbf{K}}\left(\mathbf{r}\right)=\delta {\tilde{m }}_{\mathbf{K}}\left(\mathbf{r}\right){e}^{i\mathbf{K}\cdot \mathbf{r}}$$

being $$\delta {\tilde{m }}_{\mathbf{K}}\left(\mathbf{r}\right)=\left(\begin{array}{c}\mathrm{sin}{\theta }_{k}\delta {\varphi }_{k}\\ \delta {\theta }_{k}\end{array}\right)$$ the cell function of the dynamic magnetization, and $\mathbf{r}$ the generic position vector, which, in the discretized periodic sample, depends on the micromagnetic cell index $k$ (going from 1 to N in the primitive cell), and possibly includes a lattice vector $\mathbf{R}\mathbf{^{\prime}}$ to span across the full periodic system, i.e., $\mathbf{r}\left(x,y,z\right)={\mathbf{r}}_{k}+\mathbf{R}\mathbf{^{\prime}}$. The computation and diagonalization of the dynamical matrix, out of any equilibrium magnetization distribution, is performed by an appropriate software, developed in parallel with the theoretical model at the University of Ferrara.
